# SOCS1 deficiency—crossroads of autoimmunity and autoinflammation—two case reports

**DOI:** 10.3389/fped.2024.1516017

**Published:** 2025-01-07

**Authors:** Kajetan Trojovsky, Maximilian Seidl, Florian Babor, Stephan Ehl, Min Ae Lee-Kirsch, Michael Friedt, Hans-Juergen Laws, Nibras Naami, Prasad Thomas Oommen, Sujal Ghosh

**Affiliations:** ^1^ Department of Pediatric Oncology, Hematology and Clinical Immunology, Medical Faculty, Center of Child and Adolescent Health, Heinrich-Heine-University and University Hospital, Duesseldorf, Germany; ^2^ Institute of Pathology, Heinrich Heine University and University Hospital of Duesseldorf, Duesseldorf, Germany; ^3^ Center for Chronic Immunodeficiency, Faculty of Medicine, University of Freiburg, Freiburg, Germany; ^4^ Center for Pediatrics and Adolescent Medicine, Medical Center, Faculty of Medicine, University of Freiburg, Freiburg, Germany; ^5^ Department of Pediatrics, Medizinische Fakultät Carl Gustav Carus, Technische Universität Dresden, Dresden, Germany; ^6^ Department of General Pediatrics, Neonatology and Pediatric Cardiology, Division of Pediatric Gastroenterology, Heinrich-Heine-University and University Hospital, Duesseldorf, Germany; ^7^ Department of Pediatric Oncology and Hematology Herdecke, University Hospital Witten/Herdecke, Herdecke, Germany

**Keywords:** SOCS1, SOCS1 haploinsufficiency, SOCS1 deficiency, CRMO, CNO, autoinflammation, autoimmunity

## Abstract

Suppressors of cytokine signaling (SOCS) proteins play a critical role in regulating immune signaling pathways. Deficiency of SOCS1 leads to various autoimmune pathologies. We present two unrelated patients with distinct clinical manifestations. Patient 1, a 16-year-old male from Guinea, presented with Evans Syndrome, musculoskeletal pain and elevated liver enzymes. Patient 2, a 6-year-old German boy, developed recurrent oral aphthous ulcers, mild inflammatory bowel disease and chronic recurrent multifocal osteomyelitis. Both patients were diagnosed with SOCS1 deficiency by genetic testing. Treatment strategies included steroids, JAK inhibition and colchicine. These cases emphasize the importance of considering SOCS1 deficiency in patients with autoimmune or autoinflammatory diseases but also in patients with unexplained elevated IgE levels. They highlight the need for further research in ongoing multicenter registries to better understand this condition.

## Introduction

The family of suppressors of cytokine signaling (SOCS) proteins are considered as essential key regulators in IFN type I and II signaling. SOCS proteins act as negative regulators of intracellular Janus kinases (JAK), which lead to inhibition of the JAK/STAT pathway (1–3). SOCS1 in particular, has the ability, not only to help to catalyze the ubiquitination of signaling intermediates, but also to directly inhibit the JAK protein activity, predominantly the activity of JAK-2 (1, 4). Interestingly, SOCS1 also acts as a tumor suppressor and is therefore silenced in many different tumor entities (5, 6).

SOCS1 deficiency or haploinsufficiency is a rare condition, first described in human in 2020. It leads to a variety of autoimmune pathologies (7–9). The phenotypical appearance can vary considerably, even among patients harboring identical mutations (8).

In this report, we describe two unrelated cases with distinct clinical manifestations of SOCS1 deficiency.

## Case descriptions

**Figure 1:**
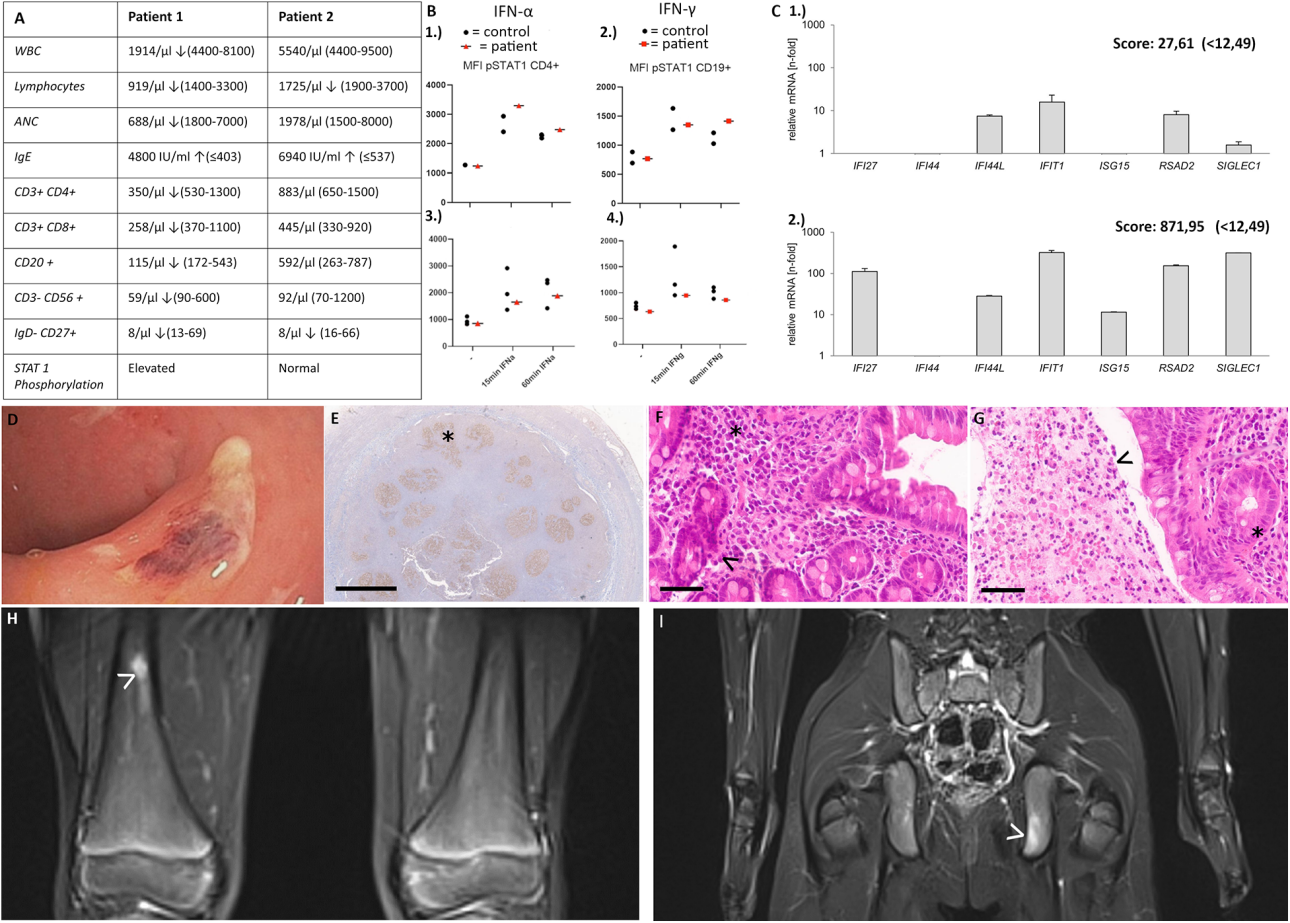
**(A)** Immunological features in patient 1 and patient 2 **(B)** STAT1 phosphorylation 1.) patient 1: elevated STAT1 phosphorylation after stimulation with IFN alpha and 2.) elevated STAT1 phosphorylation after 60 min of stimulation with IFN gamma 3.) patient 2: no elevated STAT1 phosphorylation after stimulation with IFN alpha and 4.) IFN gamma **(C)** 1.) interferon signature of patient 1 before start of ruxolitinib [slightly elevated, score 27,61 (<12,49)] 2.) interferon signature of patient 1 after 7 months of ruxolitinib treatment [strongly elevated, score 871,95 (<12,49)]. **(D–G)** Patient 2 **(D)** unspecific colitis with fibrin coated erosions in the rectum **(E)** Appendix at the age of 5 years showing irregular and progressively transformed germinal centers (BCL6 immunohistochemistry with DAB-based brown coloration, altered germinal center marked by asterisk) **(F)** Duodenum at the age of 8 years showing slightly elevated plasma cell densities in the lamina propria (asterisk), and slightly hyperplastic crypts (arrowhead) as signs of a minor, non-specific chronic inflammation. No villous atrophy and no intraepithelial lymphocytosis. **(G)** Rectum at the age of 8 years showing slightly hyperplastic mucosa on the right side, necrotic debris with neutrophils and macrophages on the left (pseudomembrane, arrowhead). Magnifications were as follows: **(E)** 1.4×, bar 2 mm; (F/G) 40×, bar 50 µm. Histological sections were digitized with the Aperio slide scanner (Leica, Nussloch, Germany), with an original magnification of 40× and an original resolution 0.253 µm per pixel. (H/I)Patient 2: Focal T2w signal alterations (arrowheads) in the distal third of the right femur **(H)**, and in the ischium on the left **(I)** indicating lesions suggestive of CRMO.

Patient 1, a 16-year-old young boy, newly migrated from Guinea, presented with neutropenia, low platelets, constipation and chronic musculoskeletal pain in shoulders and hips when he was first admitted to our hospital. Physical examination revealed splenomegaly. Whole body MRI and sonography did not reveal any features explaining the pain, however heterogeneous liver tissue suggested a history of hepatitis*.* Further investigations showed elevated liver enzymes and IgE levels, along with low memory B cells and mild T cell lymphopenia. Testing for hepatotropic viruses, autoimmune hepatitis, parasites and allergic history remained negative. “Evans syndrome” was diagnosed with positive anti-granulocyte and anti-platelet antibodies. Genetic panel sequencing covering inborn errors of immunity revealed a c.214_215delinsAA (p.A72 K) variant in *SOCS1* indicating SOCS1 deficiency. Family testing was not possible as other family members were not available. T cell proliferation was normal (PHA, anti-CD3, anti-CD3/28), however we observed a slightly elevated interferon signature with a score of 27,61 (<12,49) as expression of interferon expressing genes (IFI27, IFI44, IFI44l, IFIT1, ISG15, RSAD2, SIGLEC1) and elevated STAT1 phosphorylation (Figure 1) (10). Due to insufficient pain control with metamizole and NSAID we started treatment with steroids, providing partial pain relief and normalization of ANCs. Due to adverse effects steroids had to be tapered. Subsequent treatment with ruxolitinib (up to 2 × 20 mg) improved pain control and overall well-being but had limited impact on blood counts. Paradoxically, ruxolitinib led to a strongly increased interferon signature with a score of 871,95 (<12,49).

Patient 2, a 6-year-old German boy, was referred to our department due to recurrent oral ulcers, diarrhea and recurrent upper respiratory tract infections. His medical history included suspected milk protein allergy, appendectomy at 5 years, atopic dermatitis since infancy, recurrent episodes of epistaxis and skin infections (11). Family history revealed a maternal grandmother with bronchial asthma and recurrent upper respiratory tract infections, arthrosis and type 2 diabetes. Oesophagogastroscopy and ileocolonoscopy in our patient showed unspecific colitis with fibrin coated erosions in the rectum, as well as distal gastritis with small, aphthous ulcers in the antrum and signs of proximal duodenitis (Figure 1). Histology showed a higher inflammation intensity in the large bowel. The inflammatory pattern shared some similarities with graft-vs.-host disease, and did not resemble ulcerative colitis or Crohn‘s disease. Histology revealed an inflammatory infiltrate dominated by (neutrophil) granulocytes in earlier, a lymphocytic pattern in the latest biopsy. There were no signs of a disturbed plasma cell maturation or homing. However, germinal centers were ill-defined throughout all biopsies. (Figure 1) Colchicine was initiated and led to complete resolution of oral aphthae. Furthermore, mesalazine led to improvement of diarrhea and lasting continuous remission, also histologically.

At the age of 9 years, the patient complained about progressive limb and neck pain, with MRI findings suggestive of chronic recurrent multifocal osteomyelitis (CRMO) in various bones. Treatment with NSAID significantly reduced pain and osseous inflammation.

Due to the unusual manifestations an inborn error of immunity was suspected. Further investigations revealed elevated IgE levels and low memory B cells (Figure 1), prompting whole exome sequencing. A heterozygous deletion in *SOCS1* was identified. Further functional tests showed no abnormalities in STAT1 phosphorylation or T cell proliferation, and screening for auto-antibodies remained negative. Currently mesalazine and naproxen are well controlling the patients’ symptoms.

## Discussion

Consistent with previous clinical cases of SOCS1 deficiency, our patients presented with a variety of autoimmune and autoinflammatory symptoms (8, 9, 11–14).

Notably, patient 1 experienced musculoskeletal pain without correlating MRI abnormalities, a novel observation in this context. Treatment with ruxolitinib improved pain control and quality of life, implicating underlying inflammation, despite no significant improvement in blood counts. Interestingly, treatment with ruxolitinib led to further increased interferon signature and liver enzymes, which might be conflicting for long term usage.

Patient 2 initially presented with gastrointestinal symptoms, without histological resembling of Crohn's disease, which has been observed in SOCS1 deficiency (8, 13). Additionally, this patient represents the first case of CRMO in SOCS1 deficiency. As treatment with colchicine, mesalazine, and naproxen was effective, we did not further intensify treatment. Interestingly, the complete deletion of SOCS1 as seen in this patient, does not appear to necessarily lead to more severe clinical features (13).

Our cases highlight the importance of screening patients with autoimmune or autoinflammatory diseases for SOCS1 deficiency (11, 13, 15). Furthermore, both patients showed highly elevated IgE levels exceeding the extent of atopy seen at the time of investigation. Further studies are needed to elucidate the potential link between autoimmune processes and elevated IgE levels in SOCS1 deficiency. Treatment strategies in our patients included symptom- and organ-orientated mediation. Given the paradox results of interferon signature in patient 1, targeted JAK-Inhibition with ruxolitinib needs further investigation.

It remains of paramount importance to include these patients in multicenter registries, as the ESID SOCS1 registry, to gain more structured data regarding complications, treatment strategies and long-term outcome.

## Data Availability

The original contributions presented in the study are included in the article/Supplementary Material, further inquiries can be directed to the corresponding author.
